# Age-Related Differences in the Evaluation of a Virtual Health Agent’s Appearance and Embodiment in a Health-Related Interaction: Experimental Lab Study

**DOI:** 10.2196/13726

**Published:** 2020-04-23

**Authors:** Carolin Straßmann, Nicole C Krämer, Hendrik Buschmeier, Stefan Kopp

**Affiliations:** 1 Computer Science Institute University of Applied Sciences Ruhr West Bottrop Germany; 2 Social Psychology: Media and Communication University Duisburg-Essen Duisburg Germany; 3 Social Cognitive Systems Group CITEC Bielefeld University Bielefeld Germany

**Keywords:** virtual health advisor, age-related differences, human-agent interaction, appearance, embodiment

## Abstract

**Background:**

Assistive technologies have become more important owing to the aging population, especially when they foster healthy behaviors. Because of their natural interface, virtual agents are promising assistants for people in need of support. To engage people during an interaction with these technologies, such assistants need to match the users´ needs and preferences, especially with regard to social outcomes.

**Objective:**

Prior research has already determined the importance of an agent’s appearance in a human-agent interaction. As seniors can particularly benefit from the use of virtual agents to maintain their autonomy, it is important to investigate their special needs. However, there are almost no studies focusing on age-related differences with regard to appearance effects.

**Methods:**

A 2×4 between-subjects design was used to investigate the age-related differences of appearance effects in a human-agent interaction. In this study, 46 seniors and 84 students interacted in a health scenario with a virtual agent, whose appearance varied (cartoon-stylized humanoid agent, cartoon-stylized machine-like agent, more realistic humanoid agent, and nonembodied agent [voice only]). After the interaction, participants reported on the evaluation of the agent, usage intention, perceived presence of the agent, bonding toward the agent, and overall evaluation of the interaction.

**Results:**

The findings suggested that seniors evaluated the agent more positively (liked the agent more and evaluated it as more realistic, attractive, and sociable) and showed more bonding toward the agent regardless of the appearance than did students. In addition, interaction effects were found. Seniors reported the highest usage intention for the cartoon-stylized humanoid agent, whereas students reported the lowest usage intention for this agent. The same pattern was found for participant bonding with the agent. Seniors showed more bonding when interacting with the cartoon-stylized humanoid agent or voice only agent, whereas students showed the least bonding when interacting with the cartoon-stylized humanoid agent.

**Conclusions:**

In health-related interactions, target group–related differences exist with regard to a virtual assistant’s appearance. When elderly individuals are the target group, a humanoid virtual assistant might trigger specific social responses and be evaluated more positively at least in short-term interactions.

## Introduction

As care persons are lacking and, at the same time, most current industrial societies have an aging population, assistive technologies are of great interest [1]. Owing to their natural interface and their ability to communicate in a human-like way [2], virtual agents are ubiquitously applicable. Consequently, several authors have described the use of virtual agents as a promising approach in terms of assistive elderly care [1,3,4], especially in the area of health applications [5]. There are several groups of people in need of support, for whom cognitive limitations raise problems for mastering daily life activities. These people might have problems with regular activities, such as having meals, drinking sufficiently, taking medications, and meeting social contacts [4]. More precisely, assistive agents can provide reminders to drink or take medications, foster physical activity or social gatherings, and guide users regarding household activities [1]. This application was found to be acceptable and well usable in these kinds of target groups [4]. Within this application, the agent needs to not only process tasks correctly, but also demonstrate and use social skills [5], as it is integrated in daily life and is often used in vulnerable target groups. As a virtual agent was found to trigger social responses similar to humans [6], its appearance was found to affect the human-agent interaction regarding multiple variables [7-9]. In this regard, the appearance of the agent should match the needs of special users to achieve highly efficient assistive technology. However, regarding different experiences and expectations, the preferences and needs of target groups might broadly differ. Therefore, this study aimed to investigate target group–related differences in the perception and evaluation of a virtual assistant and its appearance in an assisted-living health-related scenario. As seniors are the main target group for which virtual assistants are highly beneficial [3,4], this study investigated age-related differences by comparing students to seniors.

Although seniors in general are more skeptical about the use of technologies, such as virtual agents [10], they seem to be less critical when asked after an actual interaction with these technologies. Prior research showed that seniors evaluated these agents more positively as compared with students in general [11]. This might be caused by fewer experiences with such technologies and by the resulting lower expectations. Thus, the following can be hypothesized: seniors will evaluate the agent and the interaction therewith more positively regarding its person perception (a), liking (b), usage intention (c), usefulness (d), and enjoyment (e) as compared with students (hypothesis 1 [H1]).

Nevertheless, as mentioned earlier, younger people are more familiar with virtual agents and their use. This familiarity with the usage of these technologies most likely enhances the perceived ease of use. Therefore, the following hypothesis can be assumed: students will rate the agent as easier to use as compared with seniors (hypothesis 2 [H2]).

To date, to the authors’ knowledge, no research has investigated the effect of appearance on the preferences of different age groups in an actual human-agent interaction. However, some research has indicated that age-related differences exist in the evaluation of an agent’s appearance. Findings with regard to e-commerce [12] report that seniors prefer an abstract appearance, as it is less distracting than a human or even a human who uses movements. The authors further showed that participants preferred an animal appearance over a human appearance, a humanoid agent was too distracting, and participants did not like technical entities to simulate a human [12]. However, the research did not systematically distinguish between different facets of appearance variables. Prior research [13] highlighted that it is important to distinguish systematically between variables and that the appearance variables species, realism, and embodiment are of specific interest. With regard to the results of Chattaraman et al [12], it is still unclear what is meant by an abstract agent. The level of abstractness can depend on the species and degree of realism. Beside these methodological inaccuracies, the findings contradict prior research [13]. Here, seniors clearly preferred humanoid and realistic agents to other species, as they were more familiar with human interaction. By contrast, they mentioned feeling stultified by nonhumanoid characters and that cartoon-stylized agents are for kids. Based on these results, it was concluded that seniors can only take humanoid agents seriously and therefore will evaluate them more positively. Additionally, Tsiouri et al [5] found in qualitative focus groups that elderly individuals prefer a realistic agent, as they want to be able to look into its eyes. This supports the findings from the study by Straßmann and Krämer [13]. The contradictions between those findings can most likely be explained through an applied context [8]. In e-commerce, the focus is on the presented product instead of personal communication and assistance, and therefore, users might prefer differently designed agents. As virtual assistance is applied in health-related domains in this research, the results of Straßmann and Krämer [13] might be better applicable. Further research [14] complements the assumptions and demonstrates that the species is more important in the evaluation process of a virtual agent’s appearance for seniors and that seniors evaluate machine-like agents less positively than humanoid ones. Based on these features, for seniors, no differences between different degrees of realism are assumed, whereas they are expected to evaluate a humanoid agent more positively. Accordingly, the following hypothesis is assumed: seniors will evaluate a humanoid agent’s appearance (regardless of its degree of realism) more positively regarding its person perception (a), liking (b), and usage intention (c) as compared with an agent having a machine-like appearance (hypothesis 3 [H3]).

Furthermore, a prior qualitative study [13] stressed that seniors prefer an embodied agent to a nonembodied agent, as they like to have something to address during an interaction. By contrast, students highlighted the advantages of a nonembodied agent, as nonembodied agents are not restricted to one device or a certain screen. Therefore, students are expected to evaluate nonembodied agents more positively than embodied agents. Thus, the following hypotheses can be formulated: seniors will evaluate an embodied agent more positively regarding its person perception (a), liking (b), and usage intention (c) as compared with a nonembodied agent (hypothesis 4 [H4]) and students will evaluate an agent represented through a voice only more positively regarding its person perception (a), liking (b), and usage intention (c) as compared with an embodied agent (hypothesis 5 [H5]).

In focused application, social processes are of high interest. Prior studies demonstrated that users show bonding with virtual agents [15,16]. In interpersonal relationships and attraction, physical attractiveness and similarity are key variables. The appearance of an agent, especially its species and realism, can affect the perceived similarity and, of course, the perceived attractiveness. Therefore, how these appearance variables influence participants’ bonding and trust needs to be investigated. Findings of prior research [13] demonstrated that seniors felt more trust toward embodied agents and bonded more with humanoid agents. In contrast, students did not mention these perceptions and seemed to try to avoid such social processes [13]. Nevertheless, according to the media equation theory [17], such processes are seen to occur automatically for all human beings. In summary, whether the appearance of a virtual agent affects social processes, such as bonding and trust, and whether these are influenced by user age should be investigated. Therefore, the following research question is posed: How are bonding and trust affected by the agent’s appearance and the users’ age group (research question 1 [RQ1])?

## Methods

### Study Design

This study aimed to investigate the effects of appearance and embodiment regarding a human-agent interaction and further examine the moderating effect of age, as a possible target group is elderly individuals in need of support and health advice. Therefore, a Wizard-of-Oz study with a 2 (age group) × 4 (appearance) between-subjects design was conducted.

### Sample

Overall, 130 people participated in this study. To investigate age-related differences, two different age groups were invited to participate in this study. In total, 84 students (mean age 23.65, SD 3.84; range 18-38 years) and 46 seniors (mean age 70.93, SD 9.05; range 51-89 years) interacted with a virtual agent and evaluated it thereafter. Both groups differed significantly with regard to age (*F*_1,129_=1732.19, *P*<.001, η_part_^2^=0.931). Unfortunately, sex was not balanced, with more women (81/130, 62.3%) than men (49/130, 37.7%) participating. However, there were no differences in sex distribution between the two age groups (χ*^2^*_1_=1.59, *P*=.21). Students and seniors were further equally distributed in all four experimental conditions (χ*^2^*_3_=0.32, *P*=.96). Nevertheless, both groups differed in their prior experiences with virtual agents (χ*^2^*_1_=31.40, *P*<.001). Among the students, many (54/84, 64%) had interacted with a virtual agent in the past; however, among seniors, few (6/46, 13%) had interacted with a virtual agent in the past, and thus, the majority of seniors had no prior experience with this technique.

### Stimulus Material

**Figure 1:**
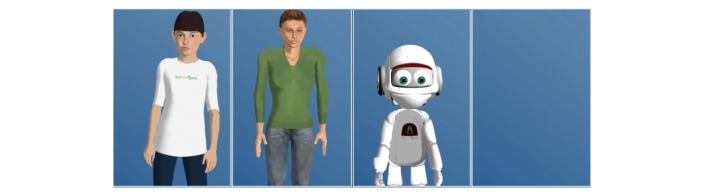
Overview of the used appearances. From left to right: Billie, Character, Vince, and Voice Only.

In this study, a real human-agent interaction was tested in a Wizard-of-Oz setting. Participants interacted with an agent that was manipulated with regard to its appearance. The behavior and interaction contents were kept constant among all conditions, and participants were randomized to one of four conditions. Owing to the between-subjects design, participants interacted with one of the following four agents differing in appearance: two humanoid agents, one machine-like agent, and one agent without embodiment (Figure 1). To investigate the effect of species in an actual interaction study, humanoid appearances (Billie and Character) and a machine-like appearance (Vince) were used. Further, the influence of realism was tested by comparing a cartoon-stylized human (Billie) and a more realistic character (Character). To investigate the effect of different degrees of realism, both appearances were chosen according to realism. Billie’s overall degree of realism was rather low, and it had a cartoon-stylized shade. Although proportions were rather natural and not stylized, the resolution was more unrealistic than realistic owing to the material and texture. In contrast, Character was characterized by a higher degree of realism, as many details were obtainable, no stylized shade or proportions were used, and the resolution was more realistic (although not completely photorealistic). Additionally, this study aimed to investigate the influence of embodiment by comparing embodied characters with a voice-only version of the agent (Figure 1).

As participants interacted with the agent, an actual agent with an underlying skeleton, which could be animated to move and talk, was needed. Owing to these technical restrictions, the possible design decisions and usable appearances were limited. Thus, the manipulation used was not as controlled as desired. Nevertheless, it is a great advantage to investigate the effect of appearance in an interaction situation, where participants are able to communicate with a virtual agent. As described earlier, appearances can be used to explore the effects of species, realism, and embodiment in an actual human-agent interaction. During an interaction, the agent used the same voice under all conditions. Its nonverbal behavior was also kept constant as much as possible.

**Figure 2:**
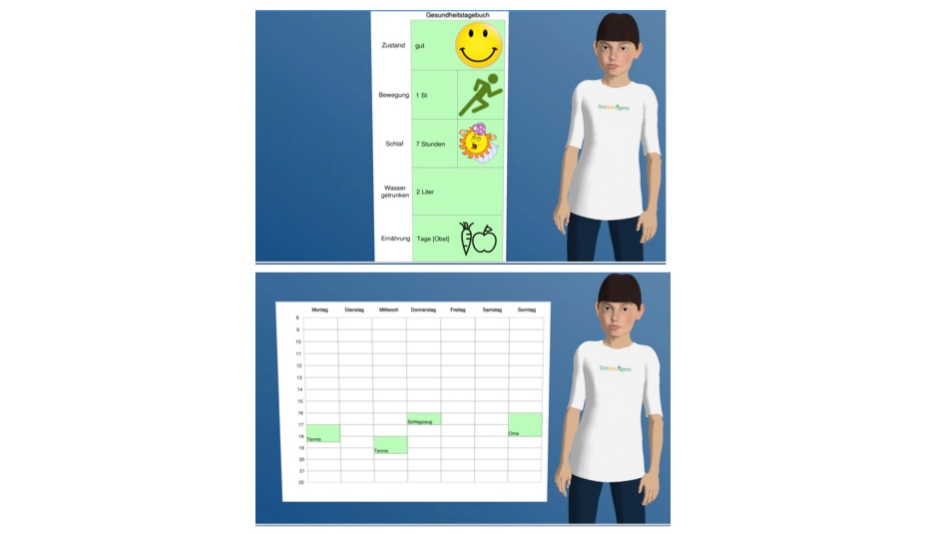
Presented tables during the interaction with the agent.

The participants’ task was to fill in a health diary and to schedule appointments in a calendar. This is a realistic and possible scenario in the application of health-related daily life assistance, as a virtual agent might be able to help with health choices, provide reminders in this regard, and help plan and structure the day. The diary entries were presented in tables and were supplemented by matching icons to adapt to people in need of support, who might have difficulties reading and understanding the textual inputs. Figure 2 presents an example of the appearance of the entries.

Overall, the interaction with the agent lasted for about 15 to 20 minutes. To guarantee a controlled and stable dialogue under all conditions, the interaction was prescripted. However, the wizard had a chance to respond to participants’ answers by including specific attributes in the prescripted dialogue (eg, the agent asked about the participants’ favorite sport and the wizard typed the answer, so that it was included in the prescripted response of the agent). Moreover, the wizard had the option to type free responses that had not been prescripted. However, this option was only used when participants digressed from the interaction topic and the wizard had to lead them back on the topic.

### Measurements

In order to examine the effect of appearance on the evaluation of the virtual agent, the person perception of the agent was assessed. Five different concepts were measured with 28 items overall on a 5-point semantic differential as follows: perceived realism (seven items, eg, “fake-natural”), likability (seven items, eg, “unfriendly-friendly”), trustworthiness (five items, eg, “not trustworthy-trustworthy”), competence (five items, eg, “incompetent-competent”), and attractiveness (four items, eg, “unattractive-attractive”). The items were adapted from prior person perception measurements [7,18]. All scales showed good reliability (Cronbach α <.808).

Supplementing this measure, participants’ liking of the agent was measured with an ad-hoc scale comprising five items (eg, “I think I would like this agent”) rated on a 5-point Likert scale (ranging from 1 [totally disagree] to 5 [totally agree]). The internal consistency of the scale was good (Cronbach α=.869).

Additionally, the usage intention of the participants was assessed. The perceived usefulness was measured with three items (eg, “I think the agent is useful to me”), and participants’ intention to use the virtual agent was measured with three items (eg, “I think I will use the agent during the next few days”). All items were rated on a 5-point Likert scale (ranging from 1 [totally disagree] to 5 [totally agree]). Both scales showed excellent internal consistency (Cronbach α <.934).

Furthermore, trust in the virtual agent was queried with two items (eg, “I would trust the robot if it gave me advice”) rated on a 5-point Likert scale (ranging from 1 [totally disagree] to 5 [totally agree]). The reliability of this scale was excellent (Cronbach α=.930).

As the relationship between user and agent is the focus of this work, participants’ bonding with the agent was measured using the bonding subscale of the Working Alliance Inventory [19]. The 12 included items (eg, “I feel uncomfortable with the agent*”*) were rated on a 5-point Likert scale (ranging from 1 [totally disagree] to 5 [totally agree])*,* and there was good reliability (Cronbach α=.842).

To evaluate the interaction, enjoyment (five items, eg, “I enjoyed the agent talking to me”), ease of use (five items, eg, “I think I will know quickly how to use the agent”), and sociability (four items, eg, “I consider the robot a pleasant conversational partner”) were measured. Participants evaluated all items on a 5-point Likert scale (ranging from 1 [totally disagree] to 5 [totally agree]). Cronbach α values demonstrated acceptable internal consistency for enjoyment and sociability (Cronbach α <.799), whereas the consistency for ease of use was not acceptable (Cronbach α=.544). Nevertheless, as this concept was of high relevance in this study, it was included in further analyses, but the results need to be discussed cautiously.

In addition, participants’ intended health behavior was measured to determine whether there was any influence by the virtual agent. This was measured with five items (eg, “Eat a well-balanced diet” and “Eat fresh fruits and vegetables”) [20]. As this scale mostly involves behaviors regarding diet, three items concerning physical exercises from Cunningham and Kwon [21] were added (eg, “I am planning to be physically active on a regular basis next week”). Participants rated all items on a 5-point Likert scale (ranging from 1 [totally disagree] to 5 [totally agree]). The reliability of this scale was good (Cronbach α=.831).

Several control variables were measured in the first part of the questionnaire. They included tendency to anthropomorphize [22] (10 items, eg, “I sometimes wonder if my computer deliberately runs more slowly after I have shouted at it”; Cronbach α=.796), prior experiences, participants’ attitude (3 items, eg, “I think it is a good idea to use the virtual agent”; Cronbach α=.752), anxiety (4 items, eg, “If I should use the virtual agent, I would be afraid to make mistakes with it”; Cronbach α=.725) [18], and negative attitudes toward virtual agents [23] (14 items, eg, “I would feel uneasy if agents really had emotions”; Cronbach α >.618). At the end, sociodemographic variables, such as age and sex, were measured.

### Procedure

When participants came into the laboratory, the experimenter welcomed them. To obtain informed consent, they were informed about the background and procedure of the study. At first, all participants filled in questionnaires about personality traits, prior experiences, and other control variables. When participants finished the first part of the questionnaire, the experimental part of the study began. Participants were asked to interact with the virtual agent in the context of a health diary. During the interaction, the experimenter left the room and participants were alone with the virtual agent. They were told that the agent interacts autonomously and can understand and react to their speech and behavior, but in fact, a Wizard-of-Oz setting was used, where a confederate (“wizard”) controlled the agent from an adjacent room. Participants were asked to start the interaction with “Hello Billie.” The wizard replied to this, and the interaction began. After the interaction with the agent was finished, the second questionnaire part was started, where dependent variables were assessed. At the end, the experimenter debriefed the participants and offered an incentive (either money or course credits).

## Results

### Assessment

This study aimed to investigate the effects of species, realism, and embodiment more closely in a real human-agent interaction. Therefore, planned contrasts were used to analyze the data with regard to specific comparisons and assumed hypotheses as follows: (1) embodiment (contrast 1), Billie, Vince, and Character versus Voice Only; (2) species (contrast 2), Billie and Character versus Vince; and (3) realism (contrast 3), Billie versus Character.

Furthermore, when significant interactions were found, according to Field [24], those effects were further investigate using simple effects, in which the effects of age groups at individual levels of the different appearances were assessed.

### Person Perception

In order to test the influence of the different appearances and age groups on the person perception of the virtual assistant, a two-way multivariate analysis of variance (MANOVA) was conducted with appearance and age group as independent variables and perceived realism, likability, attractiveness, trustworthiness, and competence as dependent variables.

Using Pillai trace, there were significant effects of age group (*V*=0.21, *F*_5,118_=6.17, *P*<.001) and appearance (*V*=0.21, *F*_15,360_=1.84, *P*<.001) on person perception.

To test the hypothesis H1a, a univariate test was performed. The results indicated that seniors and students differed significantly in their evaluation of perceived realism (*F*_1,129_=9.07, *P*=.003, η_p_^2^=0.069) and attractiveness (*F*_1,129_=21.16, *P*<.001, η_p_^2^=0.148) of the agent. Seniors evaluated the agent in general as more realistic and more attractive than did students. No significant differences between the age groups were found for likability (*F*_1,129_=1.12, *P*=.29, η_p_^2^=0.009), trustworthiness (*F*_1,129_=2.34, *P*=.13, η_p_^2^=0.019), and competence (*F*_1,129_=3.75, *P*=.055, η_p_^2^=0.030) (Table 1). Overall, the hypothesis H1a was only partly supported regarding attractiveness and had to be rejected for likability, trustworthiness, and competence.

**Table 1:** Person perception evaluation of the different age groups in general.

Variable	Score, mean (SD)		
	Seniors	Students	Overall
Realism	3.22 (1.01)	2.70 (0.81)	2.89 (0.91)
Likability	4.11 (0.76)	3.95 (0.71)	4.01 (0.73)
Attractiveness	3.79 (0.91)	3.01 (0.87)	3.29 (0.96)
Trustworthiness	3.45 (0.80)	3.19 (0.86)	3.28 (0.85)
Competence	4.13 (0.61)	3.86 (0.76)	3.95 (0.71)

Univariate tests further revealed a significant difference between appearances regarding their perceived competence (*F*_3,129_=2.98, *P*=.03, η_p_^2^=0.068). Running the planned contrast, results of the analyses showed a significant effect of embodiment (t_126_=−2.21, *P*=.03, *r*=0.19), where an embodied character was evaluated as less competent than the Voice Only condition (Table 2). Furthermore, a marginally significant effect of appearance on likability was found (*F*_3,129_=2.63, *P*=.05, η_p_^2^=0.061). Again, planned contrasts were used to explore this effect more deeply. Here, a significant difference in species was found (t_126_=−2.52, *P*=.01, *r*=0.22), where Vince was evaluated as more likable than Billie and Character (Table 2). The tests yielded no significance for realism (*F*_3,129_=2.20, *P*=.09, η_p_^2^=0.051), attractiveness (*F*_3,129_=1.99, *P*=.12, η_p_^2^=0.047), and trustworthiness (*F*_3,129_=0.60, *P*=.62, η_p_^2^=0.015).

**Table 2:** Person perception evaluation for the different appearances regardless of the age group.

Variable	Score, mean (SD)				
	Billie	Vince	Character	Voice Only	Overall
Realism	2.89 (0.90)	2.76 (0.78)	2.77 (0.84)	3.12 (1.09)	2.89 (0.91)
Likability	3.82 (0.77)	4.20 (0.58)	3.82 (0.72)	4.15 (0.78)	4.01 (0.73)
Attractiveness	3.14 (0.95)	3.49 (0.98)	3.03 (0.99)	3.47 (0.88)	3.29 (0.96)
Trustworthiness	3.16 (1.00)	3.46 (0.78)	3.21 (0.77)	3.28 (0.83)	3.28 (0.85)
Competence	3.78 (0.76)	4.04 (0.66)	3.81 (0.71)	4.18 (0.67)	3.95 (0.71)

**Figure 3:**
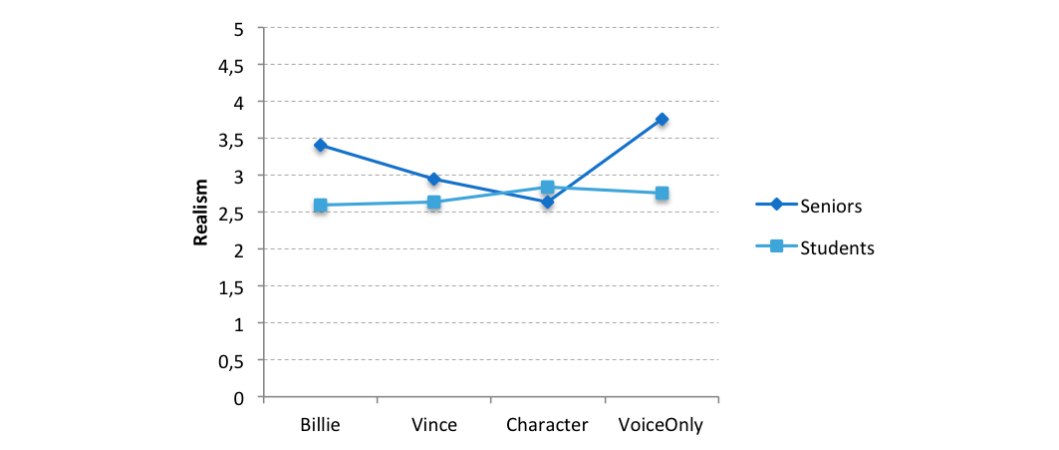
Interaction effect of appearance and age group for perceived realism. The realism scale ranged from 1 (totally disagree) to 5 (totally agree).

According to Pillai trace, there was no significant interaction effect of both dependent variables for person perception (*V*=0.09, *F*_15,360_=0.74, *P*=.75). In contrast, the univariate tests showed a significant interaction effect of appearance and age group with regard to perceived realism (*F*_3,129_=2.71, *P*=.048, η_p_^2^=0.063) but not likability (*F*_3,129_=1.18, *P*=.32, η_p_^2^=.028), attractiveness (*F*_3,129_=0.47, *P*=.71, η_p_^2^=0.011), trustworthiness (*F*_3,129_=0.73, *P*=.54, η_p_^2^=0.018), and competence (*F*_3,129_=1.33, *P*=.27, η_p_^2^=0.032). To analyze the interaction effect for perceived realism in more detail, simple effects were assessed, and it was found that seniors evaluated Billie (*F*_1,122_=6.76, *P*=.01) and Voice Only (*F*_1,122_=7.74, *P*=.002) as more realistic than did students (Billie: mean score 3.40, SD 0.83 vs 2.59, SD 0.86; Voice Only: mean score 3.76, SD 1.15 vs 2.76, SD 0.88) (Figure 3). As only an interaction for realism was found, hypotheses H3a, H4a, and H5a were not supported by the current data.

### Liking of the Agent

As effects of age and appearance on the users’ liking of the agent were assumed, a two-way analysis of variance (ANOVA) was performed with appearance and age group as independent variables and liking of the agent as a dependent variable. A significant difference between the age groups was found (*F*_1,129_=10.71, *P*=.001, η_p_^2^=0.081), where seniors liked the agents more in general than did students (mean score 3.01, SD 0.98 vs 2.39, SD 1.03). Thus, hypothesis H1b was supported. However, only a marginally significant effect of appearance (*F*_3,129_=2.41, *P*=.07, η_p_^2^=0.056) and no significant interaction effect (*F*_3,129_=0.99, *P*=.40, η_p_^2^=0.024) emerged. Planned contrasts for the appearance effect revealed an influence of species (t_126_=−2.71, *P*=.008, *r*=0.23). Users reported liking the agent more in the condition where Vince (mean score 2.93, SD 1.12) was presented than in both humanoid conditions (Billie: mean score 2.39, SD 0.92; Character: mean score 2.28, SD 0.87). In addition, interaction effects between age and appearance variables (H3–5b) were hypothesized, but the present findings did not support these hypotheses.

### Ease of Use, Perceived Usefulness, and Usage Intention

Using two-way MANOVA with appearance (four factors: Billie, Character, Vince, and Voice Only) and age group (two factors: students and seniors) as independent variables and ease of use, perceived usefulness, and usage intention as dependent variables, it was tested how the age groups differed from each other and how these variables were influenced by the appearances of different agents. Based on Pillai trace, the results indicated significant main effects for age group (*V*=0.98, *F*_3,120_=3.86, *P*=.01) and appearance (*V*=0.14, *F*_9,366_=1.98, *P*=.04) and also a significant interaction effect for both (*V*=0.16, *F*_9,366_=2.25, *P*=.02).

With regard to the univariate tests, both age groups differed in their evaluation of the agent’s ease of use (*F*_1,129_=4.07, *P*=.002, η_p_^2^=0.073), with students rating the agent as easier to use as compared with the finding for seniors (Table 3). This result is in line with the assumed hypothesis H2, where students were expected to state higher ease of use values as compared with that for seniors. Usage intention (*F*_1,129_=0.40, *P*=.59, η_p_^2^=0.002) and perceived usefulness (*F*_1,129_=0.76, *P*=.39, η_p_^2^=0.006) did not yield statistically significant effects for age differences. Thus, hypotheses H1c and H1d were not supported by the data, as students and seniors reported the same levels of perceived usefulness and usage intention.

Regarding the univariate tests, no significant differences among appearances in participants’ usage intention (*F*_3,129_=1.20, *P*=.31, η_p_^2^=0.029), ease of use (*F*_3,129_=1.50, *P*=.22, η_p_^2^=0.035), and usefulness (*F*_3,129_=1.49, *P*=.22, η_p_^2^=0.035) occurred (Table 4).

**Table 3:** Usage intention, ease of use, and perceived usefulness evaluations of the different age groups in general.

Variable	Score, mean (SD)		
	Seniors	Students	Overall
Usage intention	2.78 (1.27)	2.54 (1.27)	2.63 (1.27)
Ease of use	3.77 (0.63)	4.17 (0.69)	4.03 (0.69)
Usefulness	2.96 (1.27)	2.80 (1.25)	2.86 (1.25)

**Table 4:** Usage intention, ease of use, and perceived usefulness evaluation for the different appearances regardless of the age group.

Variable	Score, mean (SD)				
	Billie	Vince	Character	Voice Only	Overall
Usage intention	2.70 (1.25)	2.72 (1.45)	2.33 (1.26)	2.72 (1.10)	2.63 (1.27)
Ease of use	3.81 (0.65)	4.02 (0.72)	4.09 (0.64)	4.19 (0.71)	4.03 (0.69)
Usefulness	2.65 (1.32)	2.98 (1.19)	2.56 (1.27)	3.20 (1.20)	2.86 (1.25)

**Figure 4:**
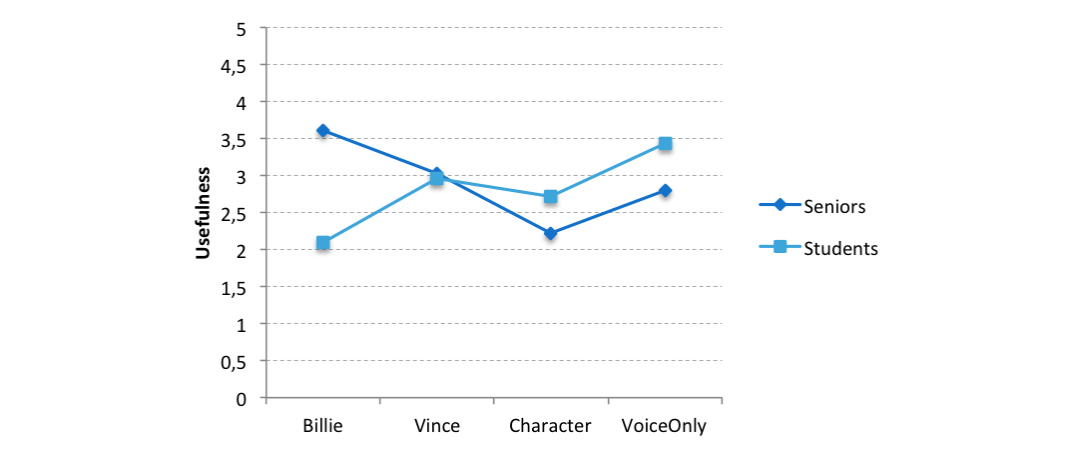
Interaction effect of appearance and age group for perceived usefulness. The usefulness scale ranged from 1 (totally disagree) to 5 (totally agree).

However, a significant interaction effect of age group and appearance was found for perceived usefulness (*F*_3,129_=5.06, *P*=.002, η_p_^2^=0.111) and usage intention (*F*_3,129_=3.38, *P*=.02, η_p_^2^=0.077) but not for ease of use (*F*_3,129_=1.96, *P*=.12, η_p_^2^=0.046) (Figure 4).

Again, simple effects were used to investigate the interaction effects. For perceived usefulness, seniors evaluated the usefulness of Billie (the cartoon-stylized humanoid agent) higher than did students (mean score 3.61, SD 1.32 vs 2.10, SD 0.97) (*F*_1,122_=12.42, *P*=.001).

**Figure 5:**
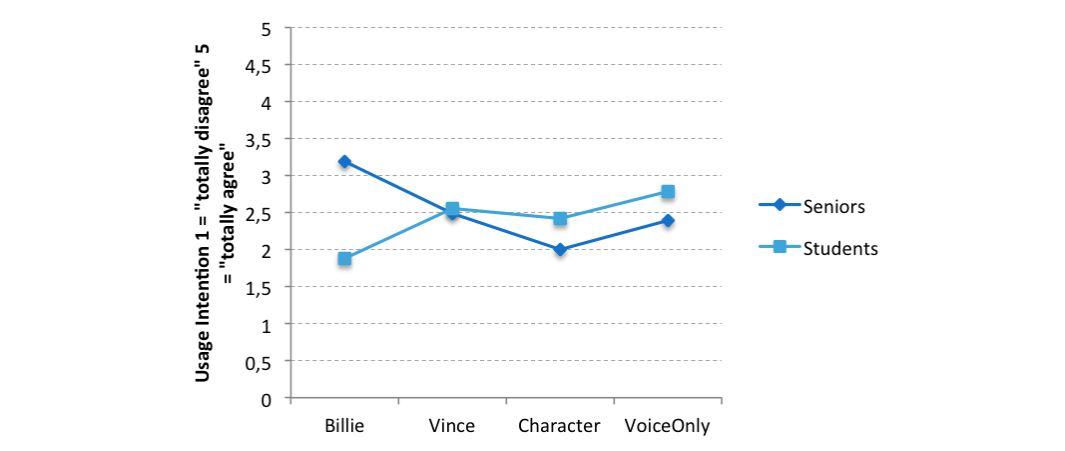
Interaction effect of appearance and age group for usage intention. The usage intention scale ranged from 1 (totally disagree) to 5 (totally agree).

In line with this finding, seniors reported higher usage intention as compared with students (mean score 3.58, SD 1.20 vs 2.19, SD 0.99) after interaction with Billie (*F*_1,122_=9.63, *P*=.002) (Figure 5).

Based on these results, the hypothesis H3b was partly supported, as it was assumed that seniors show greater usage intention for a humanoid agent. However, this was only true for a cartoon-stylized human. No such differences between both target groups were found in the evaluation of embodied and nonembodied agents; therefore, the hypotheses H4b and H5b were rejected.

### Bonding, Trust, and Sociability

As research question RQ1 aimed to investigate the effects of appearance and age group on bonding and trust, multiple two-way ANOVAs were performed to answer this research question.

The influences of age group and appearance on participants’ bonding were analyzed using two-way ANOVA with appearance (four factors: Billie, Character, Vince, and Voice Only) and age group (two factors: students and seniors) as independent variables and bonding as a dependent variable. Analyses revealed a significant main effect for age group (*F*_1,126_=11.46, *P*=.001, η_p_^2^=0.088) and a significant interaction effect for both independent variables (*F*_3,129_=3.67, *P*=.01, η_p_^2^=0.085), whereas the different appearances did not differ in participants’ bonding (*F*_3,129_=1.09, *P*=.36, η_p_^2^=0.027). Referring to descriptive values, seniors reported higher feelings of bonding toward the agent than did students (mean score 3.40, SD 0.79 vs 2.94, SD 0.67).

**Figure 6:**
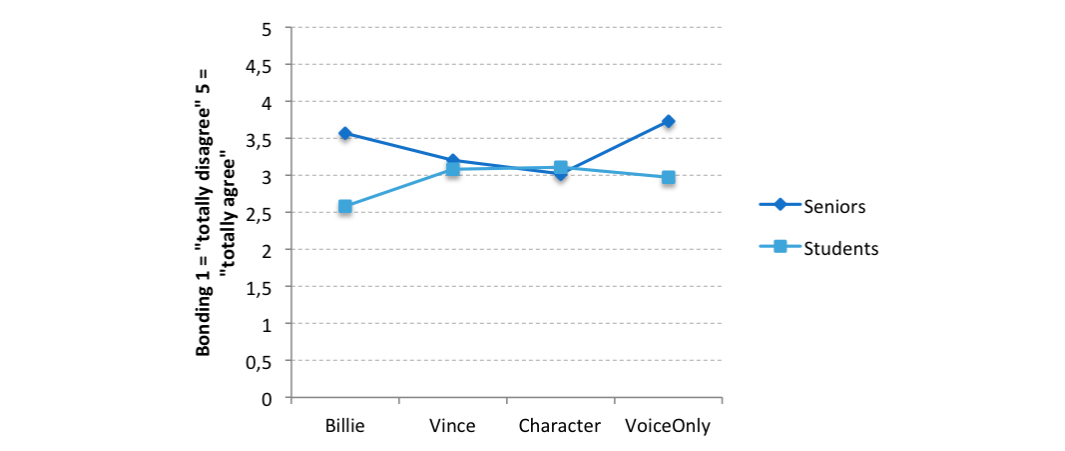
Interaction effect of appearance and age group for participant bonding. The bonding scale ranged from 1 (totally disagree) to 5 (totally agree).

To explore the interaction effect between age group and appearance further, simple effects were used. The results indicated that students and seniors showed different bonding behavior under the Billie (*F*_1,119_=13.89, *P*<.001) and Voice Only (*F*_1,122_=9.18, *P*=.003) conditions, with seniors showing higher bonding under both conditions as compared with the findings for students (Figure 6 and Table 5).

**Table 5:** Scores for all appearances among the age groups for bonding, trust, and sociability.

Variable	Score, mean (SD)							
	Billie		Vince		Character		Voice Only	
	Seniors	Students	Seniors	Students	Seniors	Students	Seniors	Students
Bonding	3.57 (0.94)	2.58 (0.45)	3.20 (0.67)	3.08 (0.75)	3.02 (0.78)	3.11 (0.76)	3.73 (0.67)	2.98 (0.58)
Trust	3.79 (0.94)	2.19 (1.04)	2.62 (1.14)	3.07 (1.28)	2.44 (1.10)	3.18 (1.16)	3.00 (1.28)	3.12 (1.13)
Sociability	4.13 (0.81)	3.00 (0.74)	4.04 (0.72)	3.81 (0.88)	3.36 (0.99)	3.44 (0.84)	4.38 (0.85)	4.05 (0.83)

**Figure 7:**
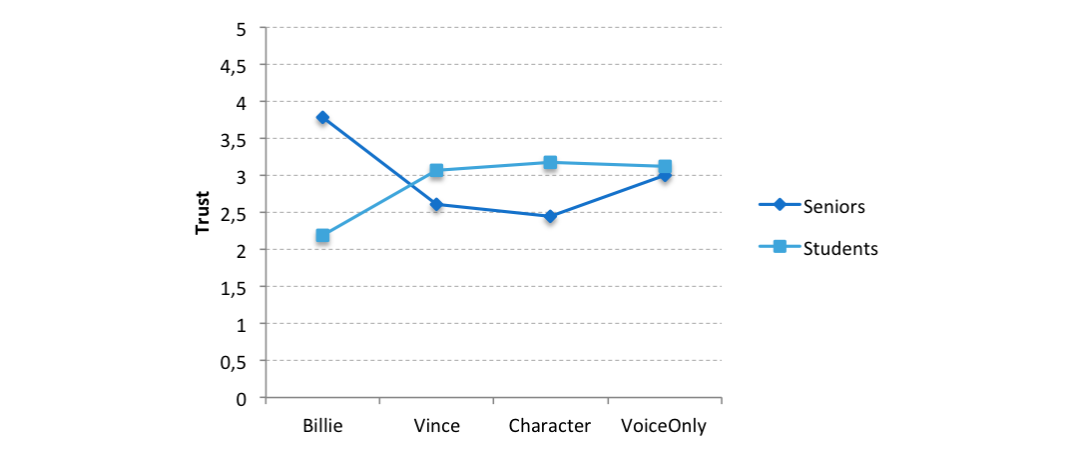
Interaction effect of appearance and age group for participants’ trust toward the agent. The trust scale ranged from 1 (totally disagree) to 5 (totally agree).

Another two-way ANOVA was performed with the same independent variables (age group and appearance) and with trust as a dependent variable. Although no significant main effects of age group (*F*_1,129_=0.13, *P*=.72, η_p_^2^=0.001) and appearance (*F*_3,129_=0.32, *P*=.81, η_p_^2^=0.008) were found for trust, the analyses yielded a significant interaction effect for both variables (*F*_3,129_=6.19, *P*=.001, η_p_^2^=0.132). Simple effects further revealed that seniors showed more trust for Billie than did students (*F*_1,122_=14.93, *P*<.001), whereas both age groups did not differ regarding trust for the other appearances (Figure 7 and Table 5).

In addition, two-way ANOVA with both independent variables and perceived sociability of the agent was performed to analyze whether the different appearances were perceived as sociable interaction partners and whether the age group had any effect. The analysis revealed a significant difference between age groups (*F*_1,129_=8.88, *P*=.003, η_p_^2^=0.068), where seniors in general evaluated the agent as more sociable than did students (mean score 4.02, SD 0.88 vs 3.53, SD 0.87).

Furthermore, a significant influence of appearance was found (*F*_3,129_=4.57, *P*=.005, η_p_^2^=0.101). The planned contrasts reveal a significant difference in perceived sociability between embodied and nonembodied agents (t_126_=−2.72, *P*=.007, *r*=0.24) and a significant difference between machine-like and humanoid agents (t_126_=−2.64, *P*=.009, *r*=0.23). Participants rated the Voice Only agent as more sociable than the three embodied agents (Voice Only: mean score 4.05, SD 0.83 vs Billie: 3.41, SD 0.93; Vince: 3.89, SD 0.82; and Character: 3.41, SD 0.87). Additionally, Vince (the machine-like agent) was found to evoke more sociability than both humanoid agents (Billie and Character).

**Figure 8:**
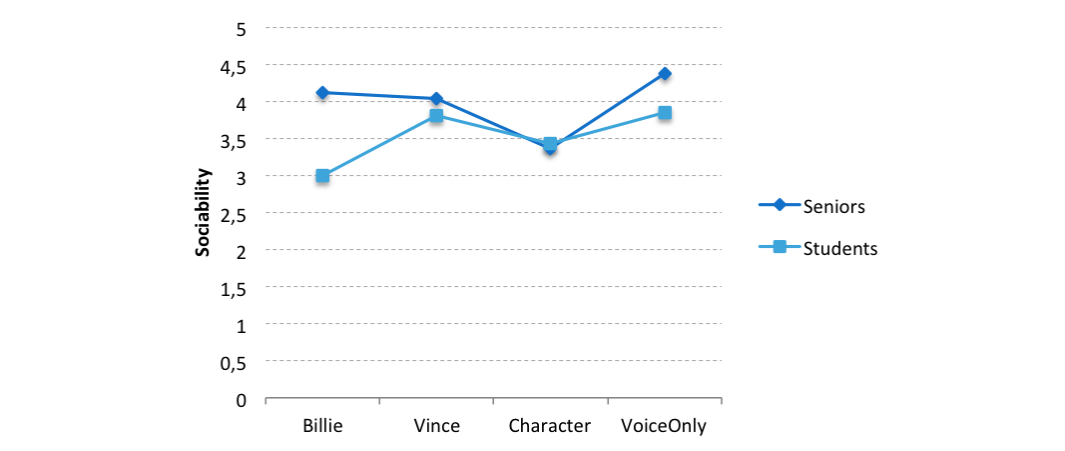
Interaction effect of appearance and age group for perceived sociability. The sociability scale ranged from 1 (totally disagree) to 5 (totally agree).

In addition, a significant interaction effect for both variables was noted (*F*_3,129_=2.80, *P*=.04, η_p_^2^=0.064). The same pattern that was found before was also obtainable for sociability, as on referring to simple effects, seniors and students differed in their evaluation of Billie (*F*_1,122_=14.48, *P*<.001) but not in their evaluation of the other appearances. Again, seniors rated Billie (the cartoon-stylized humanoid agent) as more sociable than did students (Figure 8 and Table 5), whereas no differences between both groups regarding the other appearances were noted.

### Enjoyment

As seniors are generally assumed to perceive the agent and the interaction more positively (H1), hypothesis H1e claims that seniors will enjoy the interaction with the agent more than students. The results of two-way ANOVA with age group and appearance as independent variables and enjoyment of the interaction as a dependent variable revealed a significant age effect (*F*_1,126_=7.26, *P*=.008, η_p_^2^=0.057). In line with prior findings, seniors reported the interaction as more enjoyable than did students (mean score 4.20, SD 0.82 vs 3.72, SD 0.89). Thus, the hypothesis H1e was supported by the present findings. However, the analyses yielded no significant effect for appearance (*F*_3,126_=1.61, *P*=.19, η_p_^2^=0.039) or a significant interaction effect (*F*_3,126_=2.40, *P*=.07, η_p_^2^=0.057).

## Discussion

### Results Summary and Interpretation

This study aimed to investigate age-related differences in the effects of species, realism, and embodiment on the perception and evaluation of agents in a health-related human–agent interaction, as no previous study has tested the impact of age on the perception and evaluation of appearance variables. To close this research gap, a laboratory study was conducted, in which four different appearances (cartoon-stylized human: Billie, realistic human: Character, cartoon-stylized robot: Vince, and nonembodied voice only condition: Voice Only) were tested in a between-subjects design (N=130) with two different age groups (students and seniors).

According to the findings of Rosenthal-von der Pütten et al [11], hypothesis H1 assumed that seniors will evaluate the agent and the interaction therewith more positively as compared with students. This was partly supported for person perception (H1a) and supported for liking (H1b) and enjoyment (H1e) but not for seniors’ usage intention (H1c) and perceived usefulness (H1d). In line with prior findings [11], seniors liked the agent more and enjoyed the interaction more than did students. As students probably have more experiences of interactions with virtual agents and therefore have higher and more specific expectations, it is more difficult to match their expectations and impress them. By contrast, seniors can be assumed to have mostly not interacted with a virtual agent before, and they probably have less frequent points of contact with such technologies. Therefore, they seem to have more appreciation for the technology and its functions. Additionally, the application itself is more beneficial for seniors, who need more support in their daily life. Prior research has already emphasized the influence of experience on the perceived usefulness of the technology and the intention to use it [25]. Although the interaction was designed to be suitable for both groups, the general application of daily life assistance is more adapted to people in need of support. It was therefore assumed that seniors perceive the agent as more useful and show higher usage intentions. However, these assumptions were not confirmed by the data, as no differences between the two groups were found. Potentially, social desirability influenced the seniors’ ratings. Qualitative research with elderly participants demonstrated that some elderly individuals are afraid of asking and accepting help and that elderly women especially consider the reception of help as a loss of independence [26]. Seniors (especially women, who mainly participated in this study) might want to hide their potential need for support and maintain the illusion that they do not need any help [26]. As a consequence, they might provide lower usage intention and usefulness ratings than the actual ratings. In line with this, Yaghoubzadeh et al [4] found a third-person effect [27] and reported that elderly individuals perceive virtual assistants as useful for a third person but not themselves. In summary, seniors might be afraid to be perceived as vulnerable, and therefore, they might state lower usefulness and usage intention. Another reason might lie in the fact that the seniors who participated in the study were required to visit the laboratory autonomously, which means that they needed to be mobile and healthy. Therefore, these seniors might not perfectly match the target group in need of support.

Nevertheless, with regard to the identified interaction effects, seniors rated the agent’s usefulness and their usage intention higher than did students when a specific appearance was presented. Seniors appear to perceive a cartoon-stylized humanoid agent as more useful and prefer to use an agent with this appearance.

Furthermore, in line with the hypothesis H2*,* students perceived the agent as easier to use than did seniors. This might be explained by differences in technical skills between the two groups. Students are mainly described as digital natives, who are highly familiar with the use of technology, whereas seniors are not described as digital natives. In addition, seniors’ self-efficacy with regard to these entities might be lower [28]. Users of the agent only need to talk to the agent, and therefore, the actual use of the agent should be equally easy for both groups as no technical skills are needed. Nevertheless, it has been demonstrated that seniors are more skeptical [10] and are assumed to have lower self-efficacy [28], and thus, seniors perceived the agent as less easy to use than did students.

Concerning the interaction effects of appearance variables and user age, several target group-specific differences in participants’ evaluations were assumed. Because there is scant prior research testing the preferences of senior users with regard to an agent’s appearance, according to the evidence of prior research [13,14], it was assumed that seniors prefer a humanoid appearance over a machine-like appearance (H3). Moreover, with reference to the statements in qualitative interviews [13], where seniors stated that they preferred an embodied agent because an interaction with it is more familiar and that they would like to have an interlocutor who can be addressed (eg, looking in the eyes) during the interaction, it was assumed that seniors evaluate an embodied agent more positively than a nonembodied agent (H4). In parallel, in line with students’ interview statements, the opposite was hypothesized for students, as they instead reported preferring a solely speech-based system that is more ubiquitous and not restricted to a specific screen (H5). Overall, the results showed the same pattern for multiple dependent variables. Seniors evaluated Billie (the cartoon-stylized humanoid agent) more positively than did students, whereas no differences between the target groups with regard to the other appearances were noted.

Seniors perceived Billie and Voice Only as more realistic than did students. These findings contradict the intended manipulation of realism, as it was assumed that Character would be perceived as more realistic than Billie. Prior studies have already demonstrated the importance of behavioral realism [29,30]. Accordingly, it might have been that the presented behavior of the agent matched the appearance of Billie the most and this concurrency evoked higher overall realism. However, the agent had the same behavior under all conditions, so this cannot explain the differences between the two target groups. It was shown that seniors rely more on the species of the agent and that realism is not a crucial variable for them [14]. Therefore, the differences in perceived realism might have been positive side effects of the generally more positive perception of Billie. Overall, the manipulation of both humanoid appearances might have been too subtle, so the intended manipulation was not successful. Future studies should address realism more closely.

Nevertheless, it needs to be questioned why no difference in likability or overall liking was found, although several other positive effects of a carton-stylized humanoid agent for seniors were noted. It might further be possible that participants relied more on the interaction and the agent’s behavior for their realism evaluation and that, in some way, the interactions involving Billie and Voice Only were perceived as more realistic. However, the interaction itself was designed in a maximally controlled way, where only specific prescripted answers based on a decision tree were chosen by the wizard. Nevertheless, small differences may have necessarily occurred as the agent was asked to respond to the specific answers of the participants. This limitation cannot be ruled out during an actual interaction study, where the agent should be perceived as responsive and relational. The findings cannot support the hypotheses H3a and H3b, as only differences in perceived realism and not in any other variables of the agent’s person perception were noted and no interaction effects of the target group and appearance for participants’ liking of the agent were found.

In addition, Billie was perceived as more useful and more sociable by seniors and evoked higher usage intentions, trust, and bonding in seniors as compared with students. Hence, the hypothesis H3c was supported, as seniors showed higher usage intention after the interaction with the humanoid agent Billie as compared with students. This assumption can also be extended to effects on social outcomes (RQ1). Seniors rated sociability higher for the cartoon-like humanoid agent Billie and showed more trust toward and bonding with agents with this appearance. These findings might be explained by the user groups’ expectations. Seniors might expect a virtual agent not to be photorealistic, whereas students are more used to different forms of virtual characters. Thus, it is possible that the appearance of Billie matched the seniors’ expectations the most. As predicted, there were more positive outcomes for a humanoid agent than for a cartoon-stylized one. These results indicate that regarding the application of a virtual agent in the context of daily life assistance for people in need of support, a carton-stylized humanoid appearance might be beneficial, as seniors showed, among other things, higher usage intentions, more trust, and more bonding. In the aforementioned application field, these outcomes might be very helpful, as steady intense usage of the agent is the aim. When the agent’s appearance is designed in a way that usage intention increases and when higher feelings of trust and bonding occur, regular usage behavior can be fostered. However, it needs to be acknowledged that the aforementioned results are only applicable under specific circumstances. As the findings resulted from a single short-term interaction in a Wizard-of-Oz setting, only limited deductions can be made for real-world interactions over a longer period. Because of the Wizard-of-Oz setting, the agent responded with higher accuracy than current state-of-the-art agents, which might affect the evaluation and responses toward the agent. Although the quick responses of the wizard might match the expectations participants had of the humanoid agent, when these expectations are not fulfilled in real application, people might lose interest to interact with the agent [14]. Thus, further long-term and field studies are needed to support the current findings and to derive better insights for possible design guidelines. These limitations and other shortcomings will be discussed in the next section.

### Limitations and Future Work

Although this study offered valuable insights into appearance effects within a human-agent interaction and particularly highlighted the impact of the target group, some shortcomings need to be discussed.

First, the small sample size and unbalanced distribution of age groups need to be noted. Very few seniors participated in this study, and the age cutoff was rather low for seniors (at least 50 years). Additionally, no age cutoff for students was used (studying at the university was used as a criterion for this group). During the recruitment of participants, it was aimed to maintain a balanced distribution of both groups. However, it was challenging to find elderly volunteers who could participate in a laboratory study, as they needed to be mobile and able to visit the university on their own. Nevertheless, as this study already showed the influence of the target group, studies that investigate this effect more closely with larger and more balanced samples are needed. Furthermore, other target groups that match the application field should be considered.

Moreover, the used appearances and agents had to match certain technical criteria to perform an interaction study. Therefore, the appearances could not be manipulated as systematically as desired, and they have more variance than the presented factors (species, realism, and embodiment). Although stimuli involving similar hair styles and clothing styles were chosen for the humanoid agents, they differed in not only their degree of realism but also other factors (eg, perceived age). In addition, no significant difference with regard to perceived realism was found for the realism contrast. Thus, participants appeared to perceive both humanoid agents as equally realistic. This means that the manipulation targeted needs to be evaluated with caution. Future studies should design appearances that are systematically varied and where other confounding variables are mostly eliminated. Additionally, only a limited number of appearance variables could be addressed in this experiment, and future studies should investigate the effect of other appearance variables and forms. In particular, when it comes to the realism of an agent, the uncanny valley theory [31] needs to be discussed. This research did not aim to investigate the existence of an uncanny valley, and only two stimuli that differed in realism were used. Nevertheless, as age-related differences in the evaluation and responses to different appearance variables were found, future studies should also investigate whether there are age-related differences in the perception of an uncanny valley.

In this study, no static material but an actual interaction with an agent was used. Within this interaction, the agent had behavior related to the participants’ responses. Although the interaction was scripted and the agent’s answers were limited to a certain set of possible reactions, this behavior might have influenced the interaction and the participants’ evaluation of the agent afterwards. As the interaction was aimed to be enjoyable and relatable, the agent’s answers had to rely on the prior statements of the participants. Thus, there were minor differences in the interaction and the agent’s responses. However, these minor differences cannot be prevented if an actual relational interaction is required. Behavioral realism was not the focus of this study, and therefore, it should be investigated further in the future.

Like most prior studies, this study’s results are only based on a single interaction and one point of measurement. However, the envisioned application of autonomous living and health assistance aims for a steady longitudinal usage of a virtual assistant [3]. Initial studies showed that the perception of an agent might change with time [32]. Accordingly, it still needs to be asked how the effects of appearance develop over time. Furthermore, concepts like trust and bonding do evolve over time and are more relevant in long-term interactions. Thus, long-term studies are needed to investigate the development of the presented findings in multiple interactions.

### Conclusion

This study investigated the effects of species, realism, and embodiment with regard to age-related differences on a health-related human–agent interaction. Therefore, a between-subjects laboratory study with a Wizard-of-Oz setting was used, where four different appearances and two different age groups were examined. The interaction was embedded in a virtual health assistance scenario, and participants filled in a virtual health diary together with the speech-based agent. The results emphasize the importance of the target group, as age-related differences were found in the general evaluation and in the evaluation of appearances. Seniors showed higher usage intention, trust, and bonding with a humanoid agent having cartoon stylization than did students. The realism of the agent was not found to affect the evaluation. Thus, when a virtual assistant is designed, the target group needs to be determined first. For seniors, a carton-stylized humanoid agent might be more appreciated, as it enhances usage intention and social processes. This is at least true in a short-term interaction and when a flawless interaction is provided. Overall, they appear to have a stronger need for social presence represented by a virtual human, as they are used to it from human-human communication. On the other hand, students appear not to rely on these social cues represented by appearance. This might be caused by their higher experiences with technologies and virtual agents. In summary, a health advisor for seniors should be designed with a humanoid appearance, as this fosters the interpersonal relationship and usage intention of the technology.

## Abbreviations

ANOVA: analysis of variance
MANOVA: multivariate analysis of variance
RQ: research question
